# Epidemiology of heart failure in pediatric populations in low- and middle-income countries: a protocol for a systematic review

**DOI:** 10.1186/s13643-018-0717-6

**Published:** 2018-04-02

**Authors:** Aurélie T. Sibetcheu, Valirie Ndip Agbor, Ulrich Flore Nyaga, Jean Joël Bigna, Jean Jacques Noubiap

**Affiliations:** 1 Department of Paediatrics, Faculty of Medicine and Biomedical Sciences, University of Yaoundé 1, P.O. Box 1364, Yaoundé, Cameroon; 2 Ibal Sub-Divisional Hospital, Oku, Northwest Region Cameroon; 3 Department of Internal Medicine and Specialties, Faculty of Medicine and Biomedical Sciences, University of Yaoundé 1, Yaoundé, Cameroon; 4 Department of Epidemiology and Public Health, Centre Pasteur of Cameroon, Yaoundé, Cameroon; 5 School of Public Health, Faculty of Medicine, University of Paris Sud XI, Le Kremlin-Bicêtre, France; 6 Department of Medicine, University of Cape Town, Groote Schuur Hospital, Cape Town, South Africa

**Keywords:** Epidemiology, Prevalence, Incidence, Etiology, Treatment, Mortality, Prognosis, Heart failure, Neonates, Infants, Children, Adolescents, Low- and middle-income countries

## Abstract

**Background:**

Heart failure (HF) in pediatric populations is a major public health concern. It is associated with high rates of hospital admissions, disability, and mortality in high-income countries (HIC), but its burden is poorly documented in low- and middle-income countries (LMICs). We present a protocol for a systematic review and meta-analysis to summarize available data on the prevalence, incidence, etiologies, treatment, and outcomes including hospital admission and mortality and economic burden of HF in neonates, infants, children, and adolescents in LMICs.

**Methods:**

A comprehensive search of articles published between January 01, 2000, and December 31, 2017, will be performed in PubMed/MEDLINE, EMBASE, Global Index Medicus, and Web of Science. All cross-sectional, cohort studies and case-control studies reporting on the prevalence, incidence, etiologies, treatment, prognosis, admission rates, mortality, and economic burden of HF in pediatric populations in LMICs will be included in the review. The methodological quality of included studies will be appraised accordingly. For prognosis data, the Quality in Prognosis Studies (QUIPS) tool will be used. The symmetry of funnel plot and Egger’s test will be used to identify publication bias. An overall summary estimate of prevalence/incidence of pediatric HF across studies will be obtained from study-specific estimates pooled through a random-effect model. Heterogeneity of studies will be assessed by the *χ*^2^ test on Cochrane’s Q statistic. A *p* value less than 0.05 will be considered significant for factors that predict mortality. This systematic review and meta-analysis will be reported following the PRISMA guidelines.

**Discussion:**

This study will report and summarize epidemiology data, as well as the economic burden of HF in neonates, infants, children, and adolescents of LMICs. Limitations will mainly arise from the heterogeneity in the diagnostic of HF.

**Systematic review registration:**

PROSPERO CRD42017070189.

**Electronic supplementary material:**

The online version of this article (10.1186/s13643-018-0717-6) contains supplementary material, which is available to authorized users.

## Background

Heart failure (HF) is a major threat to global public health, affecting about 26 million people worldwide [1]. Over the past decades, several cohort studies have improved our knowledge on the epidemiology of heart failure in adults. Furthermore, major advances have been done in the diagnosis and management of HF in adults, especially in developed countries. On the other hand, the global epidemiology of HF in pediatric populations remained largely unknown [2, 3]. However, it is estimated in the US that more than 14,000 hospitalizations related to pediatric HF occur annually [4], caused mainly by cardiomyopathies and congenital heart diseases (CHD). Although early operative management has improved the prognosis of CHD and prevalence of symptomatic HF to as low as 10% in some series [5], almost half of patients with cardiomyopathies develop severe and life-threatening forms of HF, necessitating cardiac transplantation [6]. Therefore, the mortality related to pediatric HF is significantly higher (more than 20 times) than in other children, reaching 7% in some studies [4]. Pediatric HF is also associated to a very high cost of care, evaluated to almost $1 billion yearly in the US, for inpatient charges only [7].

In low- and middle-income countries (LMICs), HF in adults is mainly due to hypertensive heart disease and cardiomyopathies and to a lesser degree to ischemic heart disease and valvular heart disease [8]. Available data in these countries suggest that pediatric HF is predominated by acquired and preventable causes, namely, rheumatic heart diseases (RHD), endomyocardial fibrosis, nutritional deficiencies, and other tropical diseases [9], with RHD as the first cause of cardiovascular mortality in children and young adults [10]. The main treatment option for this poverty-linked disease is surgery, especially in severe cases. Despite major advances in cardiac surgery and cardiology, most children with heart disease in LMICs are still unable to access these services, due to severely limited financial, human, and infrastructural resources [11–13]. Children of LMICs are therefore faced with a double burden of HF etiologies, added to a limited availability of resources for their management.

LMICs are characterized by a higher burden of infectious diseases, a situation that can modify etiological profile compared to other regions. Hence, streptococcal infections, the main etiology of RHD, are mainly found in Africa, Asia, Latin America, Middle East, and in the Pacific region [14]. In addition, health systems in LMICs are weak with limited access to quality health care. Cardiac surgery is almost inaccessible and not routinely performed in most of these countries.

Consequently, it is crucial to synthesize data on the magnitude of pediatric HF in order to adequately identify the challenges and gaps in the diagnosis and the management of this condition and then provide the adequate resources to curb the impact of HF on neonates, infants, children, and adolescents of LMICs. This systematic review and meta-analysis aims to summarize epidemiologic data on HF in neonates, infants, children, and adolescents in LMICs, including

- The prevalence and incidence of HF
- The etiologies of HF
- The treatment of HF and outcomes including hospital admission and mortality
- The economic burden of treatment of HF.

## Methods

### Design

This review will be conducted and reported according to the guidelines of the Centre for Reviews and Dissemination [15] and the guidelines for the Preferred Reporting Items for Systematic review and Meta-Analysis (PRISMA) [16], respectively. The present protocol conforms to the Preferred Reporting Items for Systematic review and Meta-Analysis for Protocol (PRISMA-P) [16]. An additional file shows the PRISMA for protocol checklist [see Additional file 1]. This review protocol is registered in the PROSPERO International Prospective Register of systematic reviews, registration number CRD42017070189. We do not intend to make any amendments to the protocol, to avoid the possibility of outcome reporting bias. However, amendments during the review process will be reported transparently.

### Criteria for considering studies for the review

#### Types of studies

The types of studies included are cross-sectional, case-control, or cohort studies reporting the prevalence, incidence, factors associated with, economic burden or etiologies of HF among children in LMICs, and cohort studies reporting on incidence or predictors of mortality in this population.

#### Types of participants

Studies including patients aged from 0 to 20 years will be included. If it is impossible to extract data from this age group in studies with both children and adults, these studies will be excluded.

#### Other criteria

Our search will be focused on studies published and unpublished from January 01, 2000, to December 31, 2017. We have chosen this period because the epidemiology of a disease such as HF can markedly change over time. It is therefore important to present contemporaneous data, which can be used to inform current policies and help for contemporaneous efficient strategies. Only participants from LMICs will be included as classified by the World Bank. For the 2017 fiscal year, low-income economies are defined as those with a gross national income (GNI) per capita, calculated using the World Bank Atlas method, of $1005 or less in 2016; lower-middle-income economies are those with a GNI per capita between $1026 and $3955; and upper-middle-income economies are those with a GNI per capita between $3956 and $12,2354 [17].

### Search strategy used to identify relevant studies

#### Databases searching

An expert librarian will perform a comprehensive and exhaustive search of Medline through PubMed, Excerpta Medica Database (EMBASE), Global Index Medicus (including the Literature in the Health Sciences in Latin America and the Caribbean–LILACS, Western Pacific Region Index Medicus–WPRIM, Index Medicus for the South-East Asian Region–IMSEAR, Index Medicus for the Eastern Mediterranean Region–IMEMR, Africa Index Medicus–AIM, WHO library database–WHOLIS), and Web of Science to identify all relevant articles published from January 01, 2000, to December 31, 2017, without any language restriction. A search strategy based on the combination of medical subheadings and keywords will be applied, after which the name of all LMICs will be included in the search strategy. The main search strategy for PubMed/Medline is shown in Table 1. This search strategy will be adapted to fit other considered databases.

**Table 1:** PubMed search strategy for identifying studies

Search	Query
#1	“heart failure” OR “heart fail*” OR “cardiac failure” OR “cardiac fail*” OR “cardiac insufficiency” OR “cardiac insuffic*”
#2	“Infant” OR “toddler” OR “school-age” OR “gradeschooler” OR “grade schooler” OR “grade-schooler” OR “teen” OR “teenage” OR “teenager*” OR “teen ager” OR “adolescent” OR “adolesc*” OR “Youth*” OR “child” OR “children” OR “paediatric” OR “pediatric” OR “pediatr*” OR “peaditr*” OR “newborn” OR “new born” OR “new-born” OR “neonate”
#3	Afghanistan OR Albania OR Algeria OR American Samoa OR Angola OR Argentina OR Armenia OR Azerbaijan OR Bangladesh OR Belarus OR Belize OR Benin OR Bhutan OR Bolivia OR Bosnia and Herzegovina OR Botswana OR Brazil OR Bulgaria OR Burkina Faso OR Burundi OR Cabo Verde OR Cambodia OR Cameroon OR “Central African Republic” OR Chad OR China OR Colombia OR Comoros OR “Democratic Republic of Congo” OR Congo OR “Republic of Congo” OR Costa Rica OR “Ivory Coast” OR “Cote d’Ivoire” OR Cuba OR Djibouti OR Dominica OR “Dominican Republic” OR Ecuador OR Egypt OR “El Salvador” OR Salvador OR “Equatorial Guinea ” OR Guinea OR Eritrea OR Ethiopia OR Fiji OR Gabon OR Gambia OR Georgia OR Ghana OR Grenada OR Guatemala OR Guinea OR Guinea-Bissau OR Guyana OR Haiti OR Honduras OR India OR Indonesia OR Iran. OR Iraq OR Jamaica OR Jordan OR Kazakhstan OR Kenya OR Kiribati OR Korea OR Kosovo OR Kyrgyz OR Lao PDR OR Lao OR Lebanon OR Lesotho OR Liberia OR Libya OR Macedonia OR Madagascar OR Malawi OR Malaysia OR Maldives OR Mali OR Marshall Islands OR Mauritania OR Mauritius OR Mexico OR Micronesia OR Moldova OR Mongolia OR Montenegro OR Morocco OR Mozambique OR Myanmar OR Namibia OR Nepal OR Nicaragua OR Niger OR Nigeria OR Pakistan OR Palau OR Panama OR Papua New Guinea OR Paraguay OR Peru OR Philippines OR Romania OR “Russian Federation” OR Rwanda OR Samoa OR “Sao Tomé and Principe” OR Senegal OR Serbia OR “Sierra Leone” OR Solomon Islands OR Somalia OR “South Africa” OR South Sudan OR Sri Lanka OR St Lucia OR St. Vincent and the Grenadines OR Sudan OR Suriname OR Swaziland OR Syrian Arab Republic OR Tajikistan OR Tanzania OR Thailand OR Timor-Leste OR Togo OR Tonga OR Tunisia OR Turkey OR Turkmenistan OR Tuvalu OR Uganda OR Ukraine OR Uzbekistan OR Vanuatu OR Venezuela OR Vietnam OR “West Bank and Gaza” OR Gaza OR Yemen OR Zambia OR Zimbabwe NOT (“guinea pig” OR “guinea pigs” OR “aspergillus niger”)
#4	#1 AND #2 AND #3
#5	#4 Limits: from 2000/01/01 to 2017/06/30

#### Searching for other sources

Reference lists of eligible articles and relevant reviews will be manually searched to identify other potential sources of data.

### Selection of included studies

Two review authors will independently identify articles and sequentially screen their titles and abstracts for eligibility. The full texts of potentially eligible studies will be retrieved. These review authors will further independently assess the full text of each study for eligibility and consensually retain studies to be included. Disagreements, there be any, will be solved by a third review author. A screening guide will be used to ensure that all review authors reliably apply the selection criteria. Study selection will be managed using Rayyan application for systematic reviews [18]. Studies published in English and French will be included, and eligible studies in other languages will be translated using Google Translate and considered for inclusion. Otherwise, they will be listed in the Appendix. A flowchart will be used to report the study selection process.

### Appraisal of the quality of included studies

Two review authors will independently assess study quality, with disagreements resolved by consensus or arbitration of a third review author. The methodological quality of included studies for prevalence/incidence estimate will be assessed using an adapted version of the risk of bias tool for prevalence studies developed by Hoy and colleagues [see Additional file 2] [19]. A score of 0–4, 5–7, and 8–10 will rate the risk of bias as high, moderate, and low, respectively.

The methodological quality of included studies for prognosis will be assessed using the Quality In Prognosis Studies (QUIPS) tool, designed for systematic reviews of prognostic studies through an international expert consensus [see Additional file 3] [20]. The QUIPS contains six domains assessing bias due to patient selection, attrition, measurement of prognostic factors, outcome measurement, confounding on statistical analysis and reporting results, and confounding on presentation. Hence, each included article will be scored with the algorithm developed by de Jonge and colleagues [21]. The items included in each of the six categories will be scored to evaluate the methodological quality of included studies. In case of low, moderate, or high risk of bias of individual studies, the risk of bias will be evaluated as of high (+), moderate (+/−), or low (−) quality. Each category will have a maximum score of 15 points, equally divided between all the items of each domain. The sum of each domain will give a total score, not exceeding 90 points. A score of < 54 and 54–71 (between 60 and 80% of maximum attainable score), and 72–90 (≥ 80% of maximum attainable score) will rate the risk of bias as high, moderate, and low, respectively.

### Data extraction and management

Data extraction will be conducted by two review authors, and discrepancies will be resolved through discussion and consensus or the arbitration of a third review author. The data of interest are listed in Table 2 including the outcomes. In case of missing data, the corresponding authors will be contacted to request for the missing information. We will exclude the articles from which no relevant data will be available even after contacting the authors.

**Table 2:** Data of interest

	Types of data
Study identification	Last name of the first author, year of publication, name of the journal
Study characteristics	Participants’ recruitment period, country, region, study design, study site, study setting, sampling method, timing of data collection, response rate, proportion of lost to follow-up
Participants characteristics	Mean/median age, sex distribution, proportions participants with any therapy
Diagnosis and classification	Diagnostic criteria used for HF, number of different etiologies and classification used
Prevalence, incidence, and mortality	Number of participants, total person time of follow-up, number of HF cases, number of deaths, reported etiologies, incidence rate of death in patients with HF, numbers of hospitalization, incidence rate of hospitalization in patients with HF, numbers of re-hospitalization
Economic burden data	Cost of hospital stay per patient per day, cost of medications per patient per month, cost of laboratory tests per patient per month, cost of medical devices per patient per month, cost of heart transplantation per patient, cost of surgical repair of CHD, cost of follow-up per month
Factors associated with HF and predictors of mortality, hospital admission, and readmission	As listed in studies

*HF* heart failure

### Data synthesis including assessment of heterogeneity

Data will be analyzed using R software version 3.3.3. Unadjusted prevalence and unadjusted incidence of HF and its etiologies will be recalculated based on the information of crude numerators and denominators provided by individual studies. To keep the effect of studies with extremely small or extremely large prevalence estimates on the overall estimate to a minimum, the variance of the study-specific prevalence/incidence will be stabilized with the Freeman-Tukey single arc-sine transformation before pooling the data with the random-effects meta-analysis model [22]. Heterogeneity will be evaluated by the chi-squared test on Cochrane’s Q statistic [23], which will be quantified by *I*^2^ values, assuming that *I*^2^ values of 25, 50, and 75% being the representative of low, medium, and high heterogeneity, respectively. When substantial heterogeneity will be detected (*I*^2^ > 50%), meta-regression and subgroup analyses will be performed to investigate the possible sources of heterogeneity using study characteristics, participants’ characteristics, and study methodological quality. The following subgroups will be considered: age groups (children versus adolescents), sex (female versus male), WHO regions (Africa, Americas, South-East Asia, Europe, Eastern Mediterranean, Western Pacific), and level of income (low, lower-middle, upper-middle income). Only variables significantly associated (*p* < 0.20) with the outcome of interest in univariable meta-regression analysis will be included in the multivariable model. Multivariable meta-regression analysis will be conducted if there are least ten studies per variable eligible for the model. We hypothesized that prevalence or incidence would increase when the level of income decreases. Sensitivity analysis including only studies with low risk of bias in their methodological quality will be performed. The symmetry of funnel plots and Egger’s test will be done to assess the presence of publication and selective reporting bias [24]. A *p* value < 0.10 will be considered indicative of statistically significant publication bias. The inter-rater agreement between the review authors for study inclusion and methodological quality assessment will be evaluated using Kappa Cohen’s coefficient [25]. We presume that the reporting of factors associated with HF and predictors of mortality, hospital admission, and readmission will present high reporting clinical and methodological heterogeneity. This will also be possible for other outcomes. If it is the case, we will summarize the findings in a narrative format.

## Discussion

Clinicians, researchers, and policymakers need to be informed on the situation of children presenting HF in LMICs. In order to achieve this goal, this review aims at providing estimates of the prevalence, incidence of HF, and its etiologies and prognosis, as well as the cost of care of HF among the pediatric population of LMICs. To the best of our knowledge, this will be the first systematic review and meta-analysis on the topic. The main limitation would be the ascertainment of some etiologies such as idiopathic dilated cardiomyopathy or myocarditis, which requires endomyocardial biopsy rarely done in developing countries. In addition, the limited data with a predominance of hospital-based studies could alter the generalizability of the findings. Data would also be limited with high heterogeneity on diagnostic criteria for HF, especially among children.

The current study is based on data already collected from primary studies and approved by the ethics committee, and as such, requires no ethical approval. The final report of the systematic review in the form of a scientific paper will be published in a peer-reviewed journal. Findings will further be presented at conferences and submitted to relevant health authorities. We also plan to update the review in the future to monitor changes and guide health service and policy solutions.

## Additional files

Additional file 1: PRISMA-P. (DOCX 56 kb)

Additional file 2: Risk of bias assessment tool for prevalence, incidence, and etiologies outcomes. (DOCX 13 kb)

Additional file 3: Adapted Quality in Prognosis Studies (QUIPS) list for scoring methodological quality of prognosis studies. (DOCX 14 kb)

## Abbreviations

CHD: Congenital heart diseases
HF: Heart failure
HIC: High-income countries
LMICs: Low- and middle-income countries
MOOSE: Meta-Analyses and Systematic Reviews of Observational Studies
PRISMA: Preferred Reporting Items for Systematic Review and Meta-Analysis
UK: United Kingdom
US: United States
