# Supplementary material for: Epidemiology of heart failure in pediatric populations in low- and middle-income countries: a protocol for a systematic review
Source: Syst Rev. 2018 Apr 2;7:52. doi: 10.1186/s13643-018-0717-6 (PMC5879941; doi:10.1186/s13643-018-0717-6)
Supplement: Supplementary file 2 — Risk of bias assessment tool for prevalence, incidence, and etiologies outcomes. (DOCX 13 kb) [file 13643_2018_717_MOESM2_ESM.docx]

**Additional File 2. Risk of bias assessment tool for prevalence, incidence and aetiologies outcomes**

| **Risk of Bias Item** | **Answer: Yes (Low Risk) or No (High risk)** |
| --- | --- |
| **External Validity** |  |
| 1. Was the study target population a close representation of the national population in relation to relevant variables? |  |
| 1. Was the sampling frame a true or close representation of the target population? |  |
| 1. Was some form of random selection used to select the sample, OR, was a census undertaken? |  |
| 1. Was the likelihood of non-participation bias minimal? |  |
| **Internal Validity** |  |
| 1. Were data collected directly from the subjects (as opposed to medical records)? |  |
| 1. Were acceptable case definition of condition used? |  |
| 1. Was a reliable and accepted diagnosis method utilized? |  |
| 1. Was the same mode of data collection used for all subjects? |  |
| 1. Was the length of the shortest prevalence period for the parameter of interest appropriate? |  |
| 1. Were the numerator(s) and denominator(s) for the calculation of the prevalence appropriate? |  |
| 1. Summary item on the overall risk of study bias   LOW RISK OF BIAS: 8 or more “yes” answers. Further research is very unlikely to change our confidence in the estimate.  MODERATE RISK OF BIAS: 6 to 7 “yes” answers. Further research is likely to have an important impact on our confidence in the estimate and may change the estimate.  HIGH RISK OF BIAS: 5 or fewer “yes” answers. Further research is very likely to have an important impact on our confidence in the estimate and is likely to change the estimate. |  |
