# Supplementary material for: Epidemiology of heart failure in pediatric populations in low- and middle-income countries: a protocol for a systematic review
Source: Syst Rev. 2018 Apr 2;7:52. doi: 10.1186/s13643-018-0717-6 (PMC5879941; doi:10.1186/s13643-018-0717-6)
Supplement: Supplementary file 3 — Adapted Quality in Prognosis Studies (QUIPS) list for scoring methodological quality of prognosis studies. (DOCX 14 kb) [file 13643_2018_717_MOESM3_ESM.docx]

**Additional File 3.  Adapted Quality In Prognosis Studies (QUIPS) list for scoring methodological quality of prognosis studies**

| **Domains** | **Items for consideration** | **For each item** | **Quality** |
| --- | --- | --- | --- |
| **Study participation** | a. Adequate participation in the study by eligible persons b. Description of the source population or population of interest c. Description of the baseline study sample d. Adequate description of the sampling frame and recruitment e. Adequate description of the period and place of recruitment f. Adequate description of inclusion and exclusion criteria | (+) = 3  (+/-) = 1.5  (-) = 0 | High bias: 0-6  Moderate bias: 7-12  Low bias: 13-18 |
| **Study attrition** | a. Adequate response rate for study participants b. Description of attempts to collect information on participants who dropped out c. Reasons for loss to follow-up are provided d. Adequate description of participants lost to follow-up e. There are no important differences between participants who completed the study and those who did not | (+) = 5  (+/-) = 2.5  (-) = 0 | High bias: 0-8  Moderate bias: 9-16  Low bias: 17-25 |
| **PF measurement** | a. A clear definition or description of the PF is provided b. Method of PF measurement is adequately valid and reliable c. Continuous variables are reported or appropriate cut points are used d. The method and setting of measurement of PF is the same for all study participants e. Adequate proportion of the study sample has complete data for the PF f. Appropriate methods of imputation are used for missing PF data | (+) = 5  (+/-) = 2.5  (-) = 0 | High bias: 0-8  Moderate bias: 9-16  Low bias: 17-25 |
| **Outcome measurement** | a. A clear definition of the outcome is provided b. Method of outcome measurement used is adequately valid and reliable c. The method and setting of outcome measurement is the same for all study participants | (+) = 5  (+/-) = 2.5  (-) = 0 | High bias: 0-5  Moderate bias: 6-10  Low bias: 11-15 |
| **Study confounding** | a. All important confounders are measured b. Clear definitions of the important confounders measured are provided c. Measurement of all important confounders is adequately valid and reliable d. The method and setting of confounding measurement are the same for all study participants e. Appropriate methods are used if imputation is used for missing confounder data f. Important potential confounders are accounted for in the study design g. Important potential confounders are accounted for in the analysis | (+) = 5  (+/-) = 2.5  (-) = 0 | High bias: 0-12  Moderate bias: 13-24  Low bias: 25-35 |
| **Statistical analysis and reporting** | a. Sufficient presentation of data to assess the adequacy of the analytic strategy b. Strategy for model building is appropriate and is based on a conceptual framework or model c. The selected statistical model is adequate for the design of the study d. There is no selective reporting of results | (+) = 5  (+/-) = 2.5  (-) = 0 | High bias: 0-7  Moderate bias: 8-14  Low bias: 15-20 |

PF: prognostic factor
